# Microglia in the Neuroinflammatory Pathogenesis of Alzheimer’s Disease and Related Therapeutic Targets

**DOI:** 10.3389/fimmu.2022.856376

**Published:** 2022-04-26

**Authors:** Yongle Cai, Jingliu Liu, Bin Wang, Miao Sun, Hao Yang

**Affiliations:** Institute for Fetology, The First Affiliated Hospital of Soochow University, Suzhou, China

**Keywords:** alzheimer’s disease, microglial cells, neuroinflammation, anti-neuroinflammation, molecular therapy

## Abstract

Alzheimer’s disease (AD) is the most prevalent neurodegenerative disease worldwide, characterized by progressive neuron degeneration or loss due to excessive accumulation of β-amyloid (Aβ) peptides, formation of neurofibrillary tangles (NFTs), and hyperphosphorylated tau. The treatment of AD has been only partially successful as the majority of the pharmacotherapies on the market may alleviate some of the symptoms. In the occurrence of AD, increasing attention has been paid to neurodegeneration, while the resident glial cells, like microglia are also observed. Microglia, a kind of crucial glial cells associated with the innate immune response, functions as double-edge sword role in CNS. They exert a beneficial or detrimental influence on the adjacent neurons through secretion of both pro-inflammatory cytokines as well as neurotrophic factors. In addition, their endocytosis of debris and toxic protein like Aβ and tau ensures homeostasis of the neuronal microenvironment. In this review, we will systematically summarize recent research regarding the roles of microglia in AD pathology and latest microglia-associated therapeutic targets mainly including pro-inflammatory genes, anti-inflammatory genes and phagocytosis at length, some of which are contradictory and controversial and warrant to further be investigated.

## 1 Introduction

Alzheimer’s disease (AD) is the most common neurodegenerative disease, beginning with gradual memory and cognitive impairment, abnormal behavior, and progressive social dysfunction. Pathologically, AD is characterized by severe neuronal degeneration or loss mainly resulting from excessive production of senile plaques comprising β-amyloid (Aβ) proteins, and neurofibrillary tangles formed by hyperphosphorylated tau protein deposition. Furthermore, Lewy-related pathology and presynaptic protein α-synuclein (α-syn), which are primarily involved in dementia with Lewy bodies (DLB), Parkinson’s disease (PD), and multiple system atrophy (MSA), were discovered to participate in the eliciting several parts of the pathophysiology of AD. Although compelling studies on the pathological mechanism of AD have been carried out for decades, still no effective curative treatment for AD is available. At this present, the mechanism by which drugs used in the clinical treatment of AD are mainly targeted at cholinergic neurons, eliminating or inhibiting the toxicity of Aβ or tau proteins to neurons, and reducing the oxidative stress of neurons. Unfortunately, these drugs approved by the FDA for treatment of AD are all symptomatic treatment drugs, and are still unstable in suppression of the disease process, thus reflecting the imperious demands for effective treatments. Likewise, in molecular pathology studies, more attention has not only been paid to the neuron activities, but also the effects of microglial cells, and their abnormal changes in AD are also observed. Increasing studies have evidenced that microglial-mediated neuroinflammation also plays pivotal roles in the pathogenesis of AD. Therefore, the development of drugs that target microglia may be crucial to reverse the process of AD. This article will systematically review the latest progress of the pathogenic mechanism of AD associated with microglia cells, which is likely to discover valuable AD treatment targets so as to provide a deep insight into new therapeutic approaches for AD.

## 2 The Pathological Mechanism of AD

**Figure 1:**
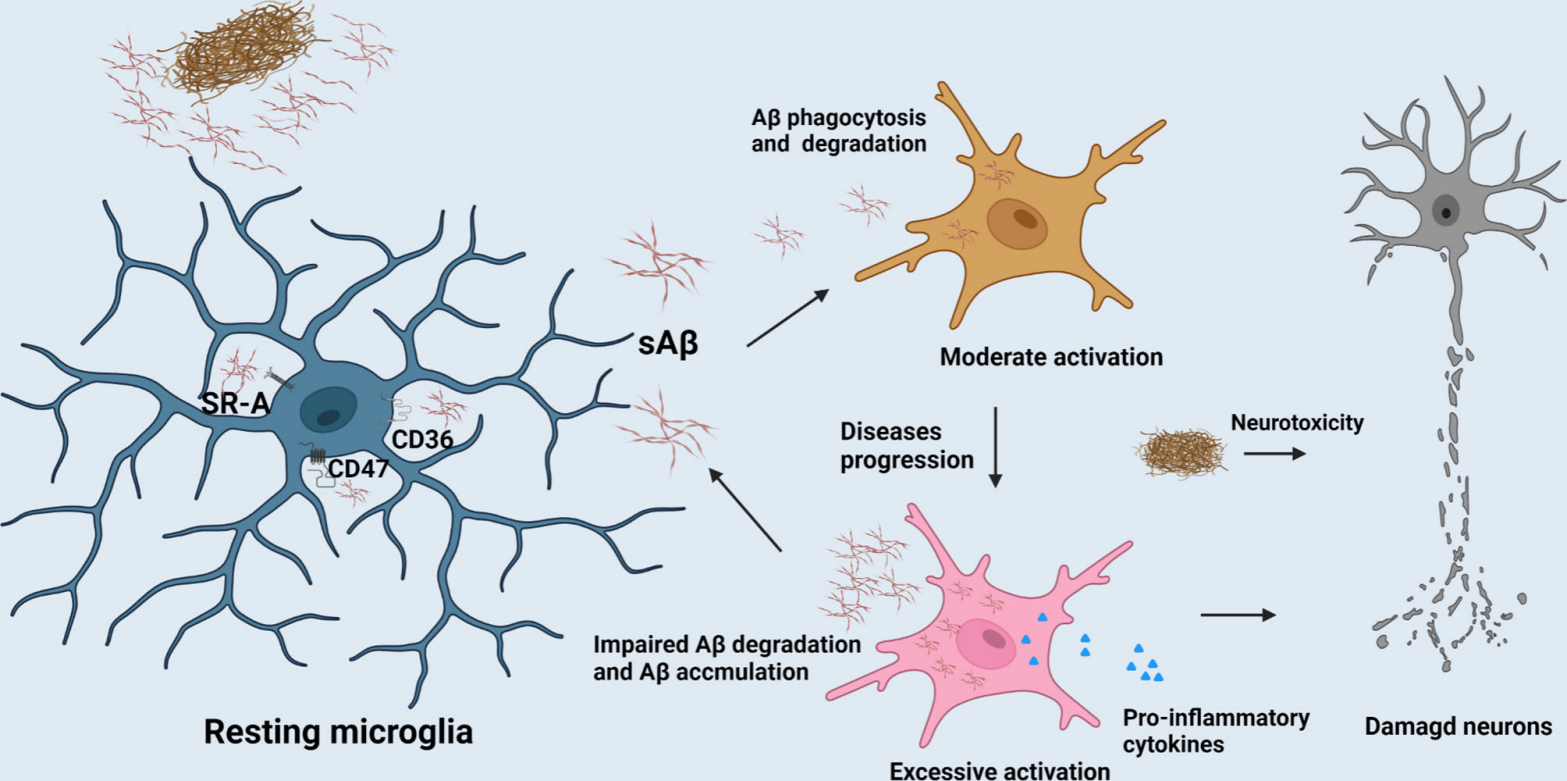
The role of microglia in AD progression. The clearance of Aβ mediated by microglia contributes to the homeostasis maintenance of CNS. But, with the AD progression, excessive activation of microglia would release excessive pro-inflammatory factors to compromise neurons and their synapses.

As the leading cause of dementia in the elderly, AD is usually characterized by memory impairment, aphasia, loss of skills and personality, and behavioral changes, etc. (1, 2). The general pathology of AD is marked by hippocampal atrophy as well as the deepening and enlargement of the cerebral sulcus. The neuroinflammatory patches, basal forebrain cholinergic neuronal loss, and glial cell proliferation constitute the main histopathological features of AD. The current prevailing view is that the amyloid plaques and neurofibrillary tangles (NFTs) are the primary pathogenic mechanism contributing to the onset of AD (3). The causal relationship between neuronal apoptosis, neurite dystrophy, and AD remains to be elucidated since the progression of neurodegeneration is a chronic event and lasts a long period of time (4). With the gradual deepening of the investigation, the researchers gained a further understanding of AD. First, Amyloid precursor protein (APP) is cleaved into Aβ peptides by γ-secretase complex and its mutation is a primary cause of the accumulation of Aβ. Second, tau protein, a component of neural cytoskeleton, plays an indispensable role in the stabilization of cytoskeleton as well as neuronal transport, and it can be phosphorylated by tau kinase, particularly GSK-3β. Subsequently, the abnormally phosphorylated tau protein gathers each other into paired helical filaments (PHF) which detach from microtubules, then these dimers assemble into oligomers, and ultimately these oligomers develop into fibrils aggregating in neurons (5). Recent studies have shown that Aβ plaques can promote the propagating and seeding of tau in a mouse model and tau antibodies help block tau propagation within AD pathology (6, 7). Strikingly, amyloid plaques were not found in the brain tissue with the Arctic or Osaka familial mutation in *APP* by positron emission tomography (PET) amyloid ligand brain scans (8). Meanwhile, NFTs were found to be irrelevant to the memory loss and neurodegeneration (9). Accordingly, neither the amyloid plaques nor NFTs alone can completely elucidate the pathogenesis of AD. In the most neurodegenerative diseases, microglia and astrocyte are high proliferative with aberrant morphology. It has been proved that microglia, working as both phagocytic cells and innate immunocyte, play a central role in pathogenic and inflammatory responses in AD, and they are thought to function in the neuroprotection of damaged neurons and maintain homeostasis. Neuronal damage was also found to be negatively correlated with microgliosis rather than increasing amyloid load (10). Intriguingly, activated microglia has been found to induce a subtype of reactive astrocytes, namely, A1 subtype which is responsible for the damage of neurons and oligodendrocytes by the secretion of pro-inflammatory cytokines including TNF, C1q and IL-1 *via* nuclear factor κB(NF-κB)-dependent mechanisms, whereas reactive astrocytes induced by M2 microglia results in the elevated secretion of anti-inflammatory factors *via* STAT6 pathway conversely (11, 12). Currently, a growing number of drugs including cholinergic drugs, anti-Aβ/tau drugs and even mitochondrial-targeted drugs, have been applied for the treatment of AD and obviously ameliorated clinical symptoms. Among them, some drugs truly exert an anti-inflammatory effect as well (13). For instance, galantamine, as a common clinical cholinergic agent for improving cognitive function in elderly Alzheimer’s patients, is found to effectively suppress the secretion of pro-inflammatory cytokines like TNF-α and IL-1β, indicating the indispensable role of anti-neuroinflammatory therapy in AD (14). Thus, we summarize microglia is central in the inflammatory pathogenesis of neurodegenerative diseases, especially AD (**Figure 1**). In this review, we outline the relationship between microglia and neuroinflammatory response in AD, as well as the definite or potential therapeutical strategies targeting microglia of AD.

## 3 The Role of Microglia in AD

### 3.1 Physiological and Pathological Functions of Microglia

Microglial cells, a kind of innate immune cells accounting for approximately 5-20% of the glial cells in the CNS, are presumed to derive from marrow myeloid progenitors produced by yolk sac in embryonic period (15, 16). They widely distribute throughout the CNS with morphological variability. Compelling studies have shown that microglial repopulation depends upon its self-renewal ability through multiple molecules, such as interleukin-1 (IL-1) and NF-κB, rather than the peripheral macrophages from bone marrow (17). Microglial cells not only play a crucial role in both innate and adaptive immune responses against pathogens, but also maintain the homeostasis of the CNS by constant surveillance of extracellular microenvironment to rapidly clear apoptotic cell remnants and other exogenous harmful objects, which facilitate neurons survival, and the proliferation and maturation of neuronal progenitor cells (NPCs) (18, 19). Nevertheless, some scholars also argued that macrophages should invade the brain tissue and differentiate into microglia ultimately through a multi-step process when a significant number of naturally dying neurons and axons are surveilled. Although microglial cells have been defined as a type of neuroglia of mesodermal origin, so far, the origin of microglial cells in the CNS is still a matter of debate. In the healthy brain, the residing microglial cells usually are in the quiescent state and are easily activated with the transformation of morphology and function following by blood-brain barrier disruption, tumor, lesions, and neurodegenerative diseases. The severity of injury determines the number of reactive microglial cells (20). Synapses play a pivotal role in neuronal circuits closely associated with neuronal communication, eliciting an appropriate reaction regarding physical activities. There is evidence that microglial cells are involved in the synaptic pruning for postnatal neural circuits by complement receptor 3 (CR3), a microglia-specific phagocytic signaling pathway, and complement cascade components including C1q and C3 (18, 21). Not only that, microglia exclusively express the receptor of CX3CL1 (the chemokine fractalkine) which was positive correlation with neuronal synaptic development and plasticity by synaptic pruning like engulfment of postsynaptic and presynaptic proteins (21, 22).

### 3.2 Microglia and Neuroinflammation

Neuroinflammation means an inflammatory reaction in the CNS including immune cells infiltration, microglial activation and pro-inflammatory cytokine release (23). An increasing number of genes involved in neuroinflammation are up-regulated in ageing brain along with cognitive decline by genome-wide gene expression studies (24). Similarly, compelling evidence suggests that neuroinflammation plays a fundamental role in the pathogenesis of AD as well, and some inflammatory mediators have been up-regulated even a longer time prior to the onset of clinical symptoms in AD. The accumulation of activated microglia around damaged areas is one of hallmarks of the neuroinflammation in AD. The prevalent view is that microglia have various states in the CNS parenchyma such as the resting (or surveying) microglia, activated microglia and phagocytic microglia (19, 25). The resting microglia typically has oval cell bodies, as well as long and numerous ramified processes (26). The microglia could be activated by pathogens and abnormally deposited proteins such as Aβ in AD. Once activated, the microglia will adopt small round soma morphology with shorter and blunter processes than their resting state, and is accompanied by the up-regulation of specific antigens. Hereafter, guided by danger-associated molecular patterns (DAMPs) or pathogen-associated molecular patterns (PAMPs), the microglia migrate to lesion regions and exhibit amoeboid morphology (27, 28). In AD, activation of microglia is triggered after binding to soluble Aβ, which is mediated by cell surface receptors such as CD36, CD47 and α-6/β-1 integrin etc. This process is followed by the stimulation of intracellular Tyr kinase-based signaling cascades which are responsible for the secretion of inflammatory molecules and accumulation of intracellular Aβ (29). Impairment of Aβ phagocytosis of microglia has been explicated by the downregulation of Aβ scavenger receptors A (SR-A), CD36 and receptor for advanced-glycosylation endproducts (RAGE) etc. The clearance of Aβ mediated by microglia contributes to the homeostasis maintenance of CNS (As shown in **Figure 1**). However, overexpression of pro-inflammatory cytokines such as IL-1β and tumor necrosis factorα (TNF-α) released from microglia in AD progression period engender the detriment of neurons (30). It is widely acknowledged that microglia are classified into various polarization states based on their immunophenotypic profiles: the M1 (pro-inflammatory or classically activated) and M2 (anti-inflammatory or alternatively activated phenotype) (31). However, there is mounting evidence that microglia are far more diverse than these two categories. Apart from the above, functions, morphology, ultrastructure, and gene and protein expression features, might be used to identify microglial subtypes. Another option is serendipitous identification, albeit non-systematic and erratic. Satellite microglia, which are identified by IBA1, CD11b, and CX3CR, are found on the axonal side of neurons in the cerebral cortex of non-human primates, and half of them extend a single process to interact with the axon initial segment. The subtypes of microglia are less ramified and have a lower level of monitoring (32). *KSPG*-microglia, a subset of ramified microglia defined by the expression of keratan sulfate proteoglycan (KSPG), are broadly distributed throughout the hippocampus, brainstem, and olfactory bulb (OB) and also express IBA1, CR3, and CD11b. The microglia mainly contribute to axonal development and cellular adhesion, and emerge in response to various stressors (33). *Hox8b*-microglia, which are mostly resident in the cerebral cortex and the OB, express the microglia markers IBA1 and Cd11b. Once *Hoxb8* is lost in the hematopoietic system, the neural circuits will be destroyed, leading to anxiety-like and abnormal grooming behavior (34). *CD11c*-microglia were uncovered predominantly in the cerebellar white matter and corpus callosum of neonatal mouse brains, and their number decreases with age. The type of microglia plays a pivotal role in myelination and neurogenesis. This is mainly due to an important source of insulin-like growth factor-1 which facilitates neural regeneration (35). Besides, there exists a new microglial phenotype called “dark microglia”. Ultrastructural analyses revealed that that the kind of microglia appear gloomy mainly due to oxidative stress profiles including an electron-dense cytoplasm and nucleoplasm. The dark microglia mainly reside in mouse hippocampus, cerebral cortex, amygdala, and hypothalamus, and participate in maintenance of the blood–brain barrier and remodeling of neuronal circuits through extensively encircling axon terminals and dendritic spines with their highly ramified and thin processes. In general, the microglial markers IBA1, CX3CR, and P2RY12 are all downregulated in dark microglia, whilst CD11b is highly expressed (18, 36). Nevertheless, M1 and M2 microglial phenotypes are still widely applied to convey its beneficial or detrimental effects under diverse states. M1 phenotype releases pro-inflammatory cytokines including IL-1β, TNF-α, IL-6 and nitrogen oxide (NOx) etc., and neurotoxic substances, which is responsible for blocking neuronal differentiation, attenuating microglial phagocytosis, as well as extracellular matrix damage through the activation of nuclear factor κB and accumulation of Aβ (37, 38). Conversely, M2 phenotype is envisioned as a type of anti-inflammatory microglia. In M2 state, the anti-inflammatory cytokines (IL-4, IL-10, etc.) and neurotrophic factors (eg., nerve growth factor (NGF)) might be released to suppress glial accumulation, protect neuronal functions and enhance NSC differentiation (31). Along with this, the anti-inflammatory or pro-inflammatory cytokines released by other cells can also elicit microglial polarization. For instance, IL-17, a cytokine mainly produced by T cells and NK cells, can trigger microglia to produce inflammatory cytokines, whereas IL-4, an anti-inflammatory cytokine, primarily produced by mature lymphoid cells and mast cells, can polarize microglia to an anti-inflammatory phenotype (39). Baik et al. found exposure to Aβ can trigger acute microglial inflammation and display a breakdown in energy metabolisms dependent on mTOR-HIF-1α pathway. Intriguingly, the inflammation caused by defective glycolytic metabolism can be reversed by IFN-γ treatment (40). Disease-associated microglia (DAM), a new type of microglia uncovered by Amit et al. provide a new pathway for the study of the pathological mechanism of AD (41). Similarly, Rangaraju et al. have revealed that DAM can also be divided into three subgroups, namely, homeostatic, pro-inflammatory and anti-inflammatory phenotypes by flow cytometry in mouse models. Pro-inflammatory DAM express IL-1β, IL-12β and surface marker CD44 etc., while anti-inflammatory DAM is characterized by phagocytic genes such as IGF-1 and surface marker CXCR4. Although the signature of DAM in 5xFAD model is not really same as that of human microglia in AD, 67 hub genes in human brain proteome were found to predominantly be mapped to the pro-inflammatory and anti-inflammatory DAM modules in their study. The top 3 pro-inflammatory DAM genes *CD44, Cst2 and Nampt* were identified in human brain proteome which are conducive to the clinical diagnosis of AD (42). Their activation can be initiated in a Trem2-independent manner that includes downregulation of microglia checkpoints (41). While, the counterpart of DAM in humans called human AD microglia (HAM) exhibits a little resemblance with DAM profile defined in AD mice in recent research. Mancuso et al. validated the differences between the responses of human and mouse microglia to oligomeric Aβ (43). Human microglia seem to display IRF8-driven gene signature once stimulated by Aβ, which is a characteristic feature in the pathology of peripheral nerve injury rather than AD in mouse models. Surprisingly, despite species differences, TREM2 is necessary in both human and mouse AD (44). Srinivasan et al. have revealed that *APOE, ABCA7, GPR141, PTK2B, SPI1* and *ZYX* etc. seemed to be upregulated, while *MEF2C* etc. remained downregulated in HAM from AD patients when compared with the control, in which, only *APOE* upregulation and *SERPINF1* reduction achieved nominal genome-wide significance after correction for multiple testing (45). These could be explained by the differences between human and mouse innate immune responses. Another explanation is that, the activation of healthy microglia is likely more beneficial in the early stage of AD models, whereas microglial activation in human AD involves impairments (15, 41). This may be justified by the fact that human AD microglia only mirror mouse microglia responses at the very early stages of the AD (46). But the most acute controversy is that human microglia live a long time, deposition of Aβ occurs over decades, and cellular functions decrease during long-term exposure to pathogens and insults in human life span. Therefore, aging, as the largest single risk factor, could not be simulated in any AD mouse models (47). This is consistent with the notion that accelerated aging response and disease specific response such as *APOE* overexpression, constitute microglial response in human AD. Intriguingly, the expression of resting microglia module defined from DAM increased in microglia from AD patients’ tissues, and HAM from high AD pathology tissues showed an increase expression in the Aging-Up gene sets. Still, human microglia do not lose the response of DAM-like manner, and this manner such as *GPNMB* upregulation is restricted in AD human brain for certain reasons (45). Olah et al. revealed the presence of nine distinct subpopulations of microglial cells purified from human cerebral cortex samples. The microglial subsets involved in homeostasis, proliferation, interferon response, and antigen presentation are obvious examples of microglial distinct subpopulations. Among a slew of genes expressed in AD genes-enriched microglial cluster 7, antigen presentation gene *CD74* were depleted in the cortex of AD patients, and only cluster 7 gene expression is altered in pathologic and dementia diagnosed human AD cortex (48). Nevertheless, some scholars believe that pro-inflammatory cytokines like IL-1β, IL-6 and TNF-α help ameliorate Aβ burden, implying that the innate immune response in AD is extremely complex and this problem still needs to be further addressed (49). In general, we hold the view that the moderate activation of microglia binding Aβ peptide can trigger neuroinflammatory reaction in the early stage of AD, which halts the progression of AD through reducing the accumulation of Aβ, and thus this activation acts as a potential neuroprotective agent. Once the sustained and immoderate production of Aβ occurred, it will result in the excessive activation of microglia, and this can exacerbate the progress of AD instead in the process of AD.

## 4 Microglial Cells-Related Therapeutic Targets in AD

### 4.1 AD-Associated Inflammatory Factors in Microglia

#### 4.1.1 NOD-Like Receptor Pyrin Domain Containing 3 Inflammation

**Figure 2:**
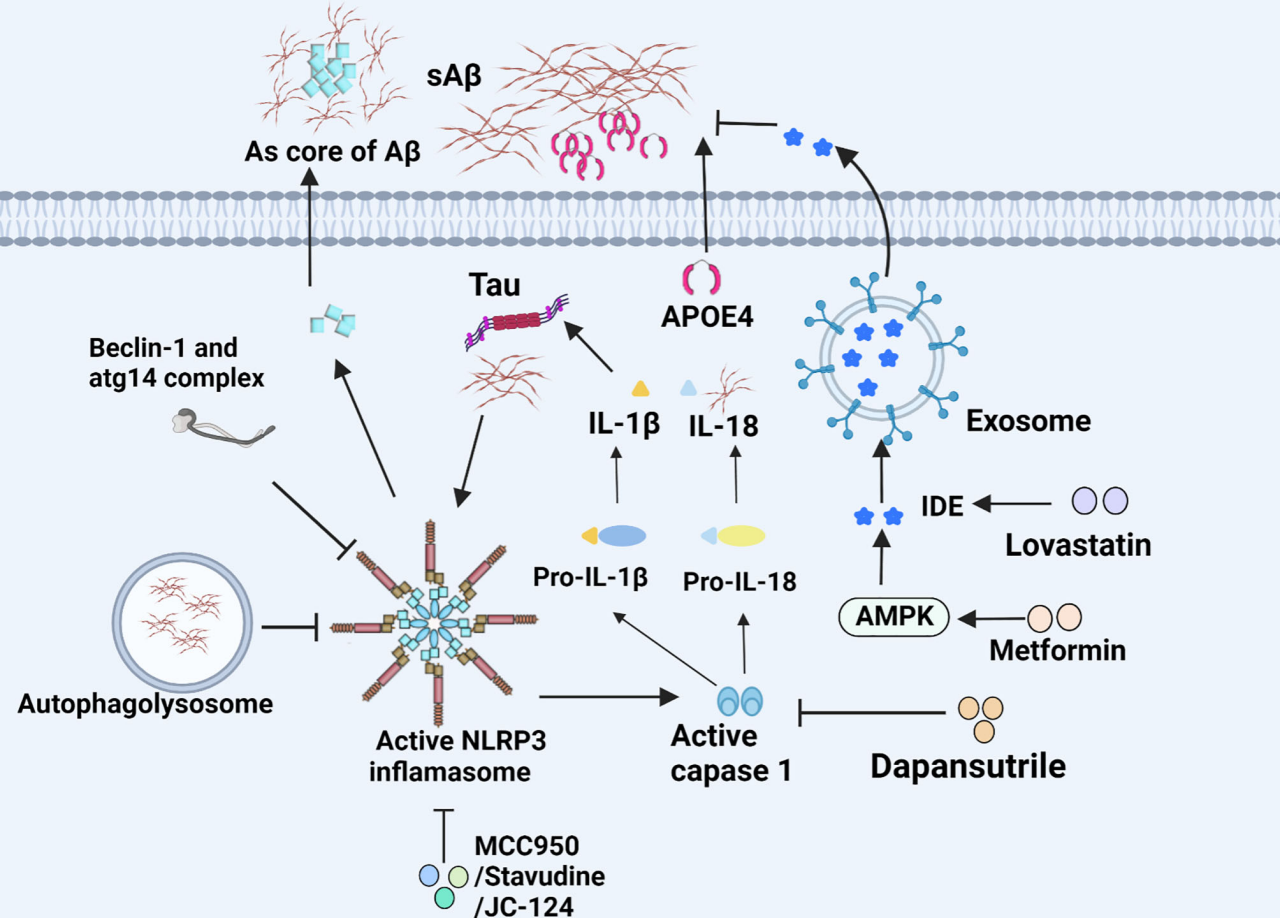
NLRP3 inflammasome and IDE in microglia of AD. The NLRP3 inflammasome accelerates microglial inflammation and can be inhibited by various substances. The secretion of IDE helps degrade Aβ and is regulated by diverse drugs.

Inflammasome, a critical component of the innate immune system, is a kind of protein complexes containing three main components, the sensor/receptor proteins, the junction protein, namely, apoptosis-associated speck-like protein (ASC), and the downstream caspase family (50). Its most well-known function is capable of mediating pyroptosis, and processing pro-IL-1β and pro-IL-18 in response to microbial infection and cellular damage through caspase-1 (51, 52). In various inflammasomes, NLRP3 inflammasome seemed more closely associated with the pathogenesis of AD such as recognizing Aβ and mediating the microglial recruitment to exogenous Aβ plaques (53, 54). Notably, *Nlrp3* ^-/-^ or *Casp* ^-/-^ mice carrying mutations associated with familial AD exhibit less complications such as the loss of spatial memory and reduce the deposition of Aβ, which demonstrate the critical pathogenesis of NLRP3/caspase-1 axis in AD (54). IL-18 facilitates the deposition of Aβ through increasing APP and altering the process of APP which were initiated by β-site APP cleaving enzyme-1 (BACE-1) and N-terminal fragment (NTF) of presenilllin-1 (PS-1). Reversely, the process of APP can be inhibited by IL-18 binding protein. Likewise, IL-1β has similar but less impact on APP compared with IL-18 (55). Intriguingly, soluble Aβ and aggregated tau activate ASC speck and NLRP3 inflammasome, leading to IL-1β secretion (56, 57). In turn, IL-1β can further upregulate ROS through transient receptor potential melastatin2 (TRPM2) pathway, indicating a vicious circle in the neuron damage of AD. Notably, MCC950, a potent and selective inhibitor of the NLRP3 inflammasome, is able to block NLRP3 inflammasome activation and reverses tau pathology when delivered exogenously (58). Currently, a number of bioactive compounds have been found to suppress the expression of NLRP3 inflammasome through multiple signaling pathways. Dapansutrile (OLT1177), an orally available small molecule inhibitor for NLRP3 inflammasome which can silence caspase-1 and IL-1β and has well tolerated and free side effects in humans, could rescue synaptic plasticity, suppress inimical microglia, and reduce the number of plaques in APP/PS1 mouse model for AD (59). Besides, there are also certain drugs in clinical use, for example, Stavudine. The drug is of great benefit to patients with AD by decreasing the expression of NLRP3 inflammasome genes to hamper the assembly of NLRP3 inflammasome, and down-regulating ERK1/2 and AKT phosphorylation to enhance Aβ autophagy (60). Another NLRP3 inflammasome inhibitor, JC-124 can selectively block the NLRP3 signaling pathway in TgCRND8 mice, subsequently resulting in reduced microgliosis, Aβ deposition, β-C-terminal fragment of APP (β-CTF), oxidative stress, and increased synaptic markers (61). Furthermore, impaired autophagy may be another pathway for NLRP3 inflammasome activation. Also, the deficiency of autophagy-related 16-like 1 (ATG16L1, an autophagy protein) and BECN1/BECLIN (an indispensable part of autophagic vesicle) actively triggers inflammasome activation, resulting in the secretion of IL-1β and IL-18 in microglia (**Figure 2**) (62, 63). In addition, the overexpression of transcription factor EB (TFEB) protein to increase LAMP1 in lysosome could effectively ameliorate autophagic activity by the downregulation of caspase-1, NLRP3, and IL-1β in BV2 microglial cells (**Figure 4**) (64).

#### 4.1.2 Apolipoprotein E

*APOE*, especially the *APOE 4* allele gene, was deemed as the critical genetic risk factor for late-onset AD, and accelerated the intraneuronal accumulation of Aβ in the brain. Compelling evidence reveals that APOE immunoreactivity widely overlaps with senile plaques (SPs) and neurofibrillary tangles, and more SPs were found in patients with *APOE 4* than *APOE 3* (65, 66). Conversely, APOE 2 acts as a protective factor against the development of AD by decreasing the sedimentation of Aβ, regulating the metabolism of lipid and maintenance of the plasticity of synapses (67, 68). In 2107, APOE was found to be abundantly expressed in microglia by Gosselin et al. (69). Next year, it was furthered revealed that the ability of microglia carrying *APOE 4* to intake Aβ was reduced, which was accompanied by the lengthening of primary processes in 3D-culture systems, implying negative correlation with its ability to phagocytose (70). In addition, APOE 4 is proved to hamper autophagy-triggered tau clearance as well. The tau-mediated neuroinflammation exacerbated by APOE 4 is identified to result in neurodegeneration independently of Aβ (71). Strikingly, Krasemann et al. also revealed that APOE signaling (not refer to APOE 4 specifically) not only suppressed the microglial homeostatic transcriptional factors, but also induced expression of inflammatory transcriptional factors such as BHLHE40, TFEC, and ATF3 etc. (4). Based on a recent study, high levels of soluble TREM2 (sTREM2) helped to ameliorate the effects of *APOE 4*-carriage on the hippocampus atrophy and cognitive decline independent of AD pathology markers in cerebrospinal fluid (72). Increasingly, JHU-083, a glutamine antagonist was found to alleviate AD pathogenesis and cognitive disorder induced by excess microglial LPS-induced glutaminase (GLS) in *APOE 4* knock-in mice (73). More interestingly, knocking down *ApoE 4* in astrocytes can rescue tau pathology and engulfment of synaptic material by microglia as well (74).

#### 4.1.3 Prostaglandin E2

**Figure 3:**
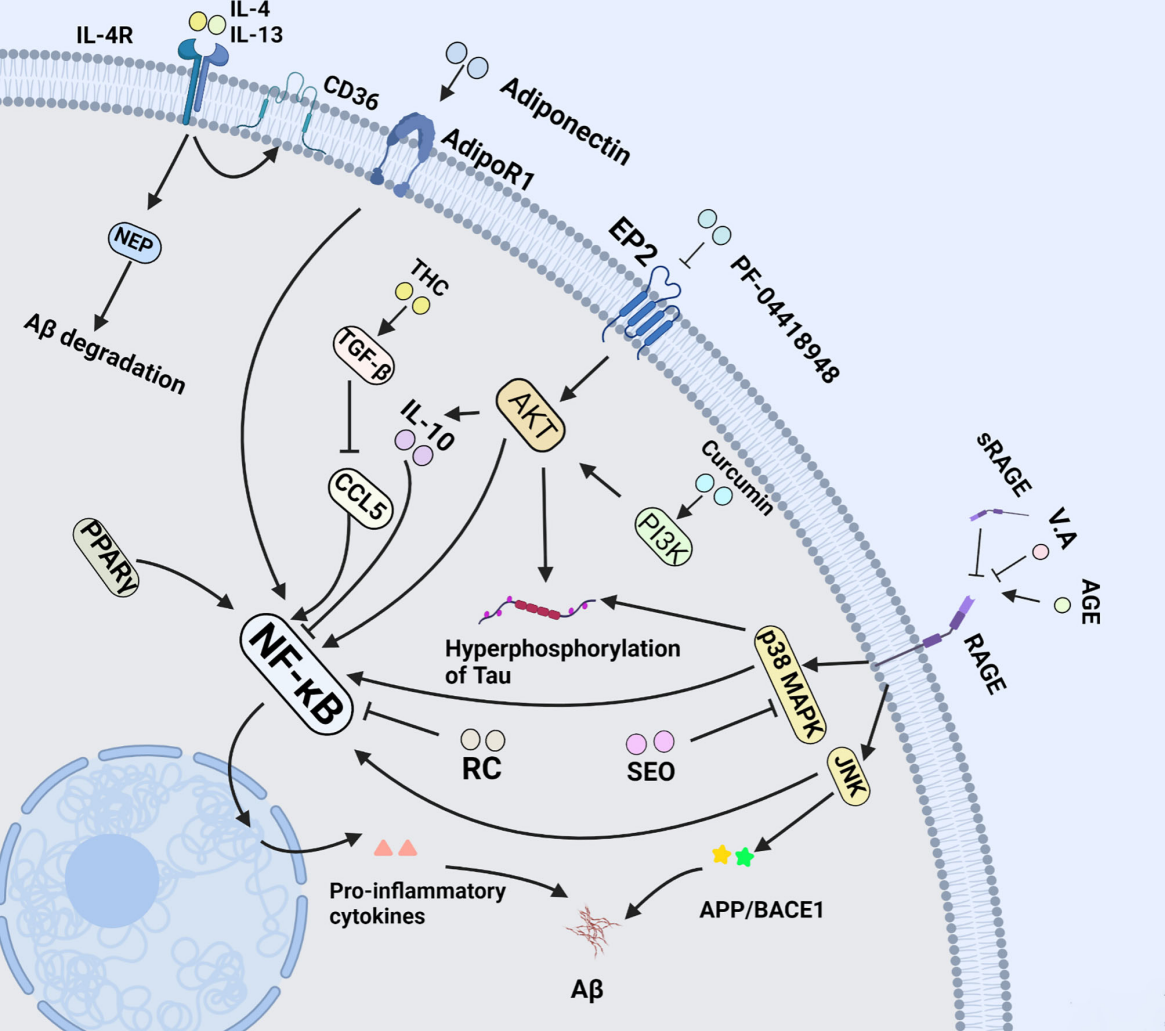
NF-κB pathway in microglia of AD. NF-κB signal pathway plays a central role in microglia-mediated neuroinflammation. This figure introduces multiple drugs mentioned in this review above, and their effects on microglia at length *via* NF-κB signal pathway.

PGE2 is the most abundant eicosanoid and acts as a kind of lipid messenger and pro-inflammatory cytokine. Its activation is associated with suppression of Aβ-stimulated microglial phagocytic activity by preventing cytoskeletal reorganization. In ageing microglia, PGE2 and its receptor EP2 can mediate the transformation of glucose into glycogen, resulting in cell energy-deficient state and pro-inflammatory responses. EP2 is emerging as a novel target for development of anti-inflammatory drugs for the treatment of chronic neurodegenerative and peripheral diseases. PF-04418948, an EP2 antagonist not only can drastically reduce pro-inflammatory cytokine in hippocampus, but also ameliorate long-term memory function *via* downstream AKT signaling pathway (**Figure 3**) (75). In addition, the deficits of novel object recognition (NOR) and spatial memory are effectively ameliorated, and the level of insulin-like growth factor 1 (IGF1) is elevated in EP2 knockout mouse model in parallel with down-regulation of pro-inflammatory factors (76). Likewise, Cyclooxygenase 2 (COX2) is a key enzyme in the synthesis process of prostaglandin E2. S-ibuprofen, a type of selective COX2 inhibitor, can rescue the phagocytic response triggered by Aβ in microglia after incubating with IL-1β overnight. Nevertheless, some specific nonsteroidal anti-inflammatory drugs (NSAIDs) like ibuprofen and indomethacin were identified to directly affect Aβ deposition by altering γ-secretase activity rather than COX activity (77).

#### 4.1.4 IL-1β and IL-6

IL-1 family proteins are mainly involved in innate immunity with a broad spectrum of diseases, and normally mediate the host response to infections, injury, and immunologic challenges. Once triggered uncontrolledly, they are more likely to produce detrimental side effects. Currently, the IL-1 family is comprised of 11 members and some of which play a dual role in inflammatory response. Among them, IL-1α is constitutively present inside normal epithelial, mesenchymal and stromal cells besides microglia, whereas IL-1β, a potent pro-inflammatory cytokine, is predominantly produced under disease conditions by macrophages/microglia, monocytes and dendritic cells (78). Biologically, IL-1 (not merely IL-1β) is selectively expressed by activated, plaque-associated microglia of brain tissue in the patients with AD (79). The pathology is initiated and driven, in part, by early and sustained overexpression of IL-1 and consequent overexpression of products of IL-1-driven cascades. The secretion of mature IL-1β needs the activation of NF-κB signaling cascades, ultimately causing the upregulation of the transcription and inflammasome to process pro-IL-1β protein (80). Apart with the above-mentioned, IL-1 is also responsible for the reactive gliosis for its stimulating other pro-inflammatory cytokines, for example, TNF-α and IL-6. Similarly, Essential oil (SEO), a drug for microbial infections and inflammation could inhibit p38 activation which results in anti-AD effects by modulating neuroinflammation through the NF-κB/MAPK signaling pathway, and ultimately improve animal cognitive capacity and histopathological changes in AD model. In addition, the anti-inflammatory effects of SEO were replicated in BV-2 microglial cells (81). Of note, Aβ in serum also triggers ATP-mediated IL-1β release from microglia (82). In contrast, several studies have revealed that the successive expression of IL-1β in the hippocampus of APP/PS1 mice decreases Aβ plaque load and enhances Aβ clearance, which mainly attributes to an increase in the ability of microglia to proliferate and the up-regulation of phagocytosis-related genes caused by IL-1β (83). To date, however the mechanisms of IL-1β-induced plaque clearance remain poorly understood.

IL-6, a prototypic cytokine is not only associated with inflammation and infection responses, but also involved in the regulation of metabolic, regenerative, and neural processes. In pro-inflammation response, IL-6 can increase the amount of hyperphosphorylated tau protein, which results in the pathogenesis of all inflammatory diseases (84). Notably, due to lack of IL-6R in microglia, the involvement of both signaling modes (trans-signaling and classical) in the biology of IL-6 is responsible for pro-inflammatory or anti- inflammatory responses. Namely, IL-6 first binds to soluble IL-6R (sIL-6R) with low affinity to form an IL-6/sIL-6R complex, and subsequently combines with gp130 on the surface of cells, which leads to a pro-inflammatory response, while IL-6 classic signaling is required for anti-inflammatory activities *via* the activation of STAT3-mediated signaling pathways (85). Regarding the IL-6-mediated activities, there is always a considerable controversy. That is to say, IL-6 has been widely regarded as a pro-inflammatory cytokine, and also has many regenerative or anti-inflammatory activities. Interestingly, a recent study showed that the repopulating microglia can stimulate the generation of neurons, modulate local microenvironment and mitigate spatial learning deficits through IL-6 trans-signaling pathway (86). Similarly, there are also some evidences that IL-6 can induce massive gliosis to suppress Aβ deposition. Although, the mechanisms underlying involvement of IL-6 are too complex and have yet to be studied, this does not abate the fact that IL-6 is still a potential target for AD.

#### 4.1.5 Interferon-γ

IFN-γ, an inflammatory cytokine mainly released by activated natural killer (NK) cells and T helper type 1 (Th1) lymphocytes, was originally assayed as an antiviral chemokine. Its activities depend largely on the constitutive expression of membrane IL-1α (78). In general, neuroinflammation driven by IFN-γ and microglial activation has been associated with neurological disease. It was reported that IFN-γ causes reduced adult hippocampal neurogenesis, behavior despair, anhedonia, and cognitive impairment by microglial priming and polarization to M1 phenotype which leads to the release of pro-inflammatory cytokines (87). There is also evidence that IFN-γ paired with ligands of Toll-like receptors (TLRs) in microglia causes neuronal network dysfunction with NO release (88). Some drugs like glycine can reverse the pro-inflammatory markers in BV-2 microglial cells treated with IFN-γ dose-dependently (89). Of importance, another study demonstrated that rhinacanthin C (RC) could inhibit IFN-γ-mediated IL-6 and TNF-α secretion, and abrogate the activation of NF-κB and ERK signaling pathway in BV-2 microglia, which is relevant to the protection of neurons and stimulation of neurite outgrowth against Aβ toxicity (23). More interestingly, a recent study demonstrated that intraperitoneal injection of IFN-γ alleviated Aβ load both in hippocampus and cortex by initiating microglial autophagy in APP/PS1 mice, which were accompanied by the rescue of cognitive deficiency and synaptic impairments (90). Therefore, targeting microglia by modulating levels of IFN-γ in the brain may be a therapeutic strategy for neurodegenerative diseases and psychiatric disorders.

#### 4.1.6 Tumor Necrosis Factor-α

TNF-α plays pleiotropic roles in CNS including tumor killer, cell proliferation and inflammatory response. Physiologically, TNF-α is mainly expressed in microglia. Once microglia are activated by Aβ, its expression will be increased. Of note, TNF-α has an intimate relationship with other cytokines since it can result in the up-regulation of pro-inflammatory cytokines IL-1 and IFNs. Strikingly, the up-regulation of TNF-α is reported to suppress long-term-potentiation (LTP) at hippocampal glutamatergic synapses associated with learning and memory. Intriguingly, this effect is effectively reversed by anti-TNF-α antibody in young *Trem2^R47H/R47H^* rats (91). In addition, TNF-α can directly stimulate Aβ deposition by the elevation of β-Site APP cleaving enzyme 1 (BACE1) expression (92). Adiponectin (APN), an anti-inflammatory and vasculoprotective adipokine, can halt or reverse the inflammatory morphological changes in microglia through inhibiting the secretion of TNF-α when co-cultured with Aβ (**Figure 3**). As a result, when cultured with APN, the viability of HT-22 neuronal cells is protected against the microglial cytotoxicity induced by Aβ (93). Besides, TNF-α can be suppressed by IL-10 *via* down-regulating the expression of nitric oxide synthetase (iNOS) and cyclooxygenase 2 (COX-2) (94). Apart from the aforementioned, flavonoids like Luteolin and Isoflavone also exert the neuroprotection in reducing TNF-α secretion from microglia, attenuating neuronal cell death (95). The evidence above shows that blocking TNF-α is more conductive to anti-neuroinflammation therapeutic strategy in AD.

### 4.2 AD-Associated Anti-Inflammatory Factors in Microglia

#### 4.2.1 Triggering Receptor Expressed on Myeloid Cells 2

TREM2, a single pass transmembrane receptor, is specially expressed on monocyte-derived dendritic cells, osteoclasts and microglia. *In vivo*, microglia that express TREM2 can increase the phagocytosis of debris from apoptotic or damaged neurons, which influences immune functions involved in inflammation, microglial proliferation, survival and cytoskeleton remodeling in the CNS (96). When TREM2 signal is activated by its adapt protein, namely, DNAX-binding protein of 12 kDa (DAP12) through immunoreceptor tyrosine-based activation motif (ITAM) signaling pathway, it will lead to greater clearance of cell debris (97, 98). It has been proved that TREM2 mediates Aβ degradation by proteasome degradation pathways and the proteasome is interconnected with lysosome through autophagosomes (99). More notably, the TREM2-haplodeficiency microglia markedly fail to cluster and insulate around Aβ fibrils and plaques, downregulate the SDF-1α/CXCR4-mediated chemotaxis, and lose the ability of nourishing axons, thus resulting in enhanced Aβ accumulation and axonal dystrophy (100). CXCR4, a chemoattractant receptor, is vital for microglial migrating towards Aβ. Activated CXCR4 helps to rescue the ability of microglial migration. As a result, it is likely to be a potential target for AD treatment as well (100). Besides, AL002c, an anti-hTREM2 agonistic mAb, provokes TREM2-mediated beneficial roles of microglia in the mice carried with the arginine-47-histidine (R47H) mutation of *Trem2* which is associated with a substantial increase of AD risk. As a result, the systemic administration of AL002c could alleviate the load of Aβ and neurite dystrophy, impact behavior of 5xFAD mice, and temper microglial inflammatory response. Although a first-in-human phase I clinical trial of AL002 demonstrated that AL002 was generally safe and well tolerated with no serious adverse effects, and can be traced by sTREM2 and sCSF-1R. There is also evidence contrary to the view that the master regulators SPI1, SMAD3 and SALL1 of the homeostatic microglia can be restored through deleting *TREM2*, leading to repression of TREM2-apolipoprotein E (APOE) signaling pathways (4, 97). Similar to the report, There is another evidence that TREM2 has multiple roles at diverse stages of AD. Wang et al. revealed *Trem2* ^-/-^ 5XFAD mice had higher Aβ burden than wild type (WT) in 8.5 month-old, but Jay et al. demonstrated that TREM2-deficient mice exhibited reduced Iba1^+^ cells, neuroinflammation and Aβ deposition at 4 months of age, indicating that TREM2 plays detrimental role at the early stages of AD and beneficial role at late stages (96, 101). Besides, Aβ is able to potentiate the interaction between TREM2 and DAP12 and bind TREM2 directly to increase the expression of pro-inflammatory cytokines including IL-6 and MIP-1α in WT microglia, while *TREM2* KO microglia show no change in response to Aβ stimulation (102). Therefore, genetic therapy targeting *TREM2* could be a potential avenue for AD therapy.

#### 4.2.2 IL-2 and IL-4 Etc

IL-2 is a 15.5 kDa cytokine that is expressed by cells, B cells, dendritic cells, eosinophils, and macrophages/microglia. It is reported that IL-2 can nourish neurons and glia in the CNS, and further promote neurite branching such as dendrite arbors and dendritic spines (103). Unfortunately, the level of IL-2 in hippocampal of the patients with AD is remarkably reduced (104). Strikingly, the administration of IL-2 *via* an adeno-associated virus (AAV)-IL-2 vector improves memory retention, synaptic plasticity, and spine density in APP/PS1E9 mice, implying IL-2 importance in AD occurrence and therapy. In addition, IL-2 serves as an immune-modulating agent for CNS (104). In recent clinical trials, administration of low-dose IL-2 has been shown to result in the expansion of regulatory T cells (Tregs) which are inflammation-resolving mediators that regulate the microglial response to Aβ deposition and facilitate in controlling inflammation and autoimmune diseases (105). Besides, low-dose IL-2 has been shown to be safe, well tolerated, and can provide immunoregulation with few side effects (106). Therefore, it is likely that the regulatory T cells stimulated by IL-2 effectively control Aβ deposition in the brain of patients with AD.

IL-4, also known as B cell-stimulatory factor-1, is a monomeric Th2 cytokine that shows pleiotropic effects during immune responses. Currently, it has been shown that IL-4 promotes cell proliferation, survival, and immunoglobulin class switch. In the CNS, IL-4 enhances polarization of microglia/macrophages from the pro-inflammatory to the anti-inflammatory subtype, and is closely associated with tissue repair from microglia/macrophages, which suppress the pathological inflammation, and elevate expression of IL-10, TGF-β and arginase-1 (107). More importantly, it has been demonstrated that the injection of IL-4 and IL-13 decreases the density of Aβ plaque in the hippocampal of APP/PS1 mice *via* the Aβ degrading enzymes neprilysin (NEP) and CD36 (108, 109). Furthermore, in the follow-up experiment, the delivery of IL-4 was proven to improve cognitive performance and alleviate tau pathology by increasing arginase-1 positive microglia cells in the 3xTg AD mice (**Figure 3**) (110).

IL-10 was first reported as an inhibitory factor of cytokine synthesis by Fiorentino et al. in 1989 (111). In the CNS, IL-10 is mainly produced by astrocytes, microglia, and neurons, and it is capable of attenuating the expression of pro-inflammatory cytokines such as IL-1β, IL-12, TNF-α, adhesion molecules, as well as co-stimulatory molecules including CD86 and CD54 (94). IL-10 plays a critical role in the regulation of immune and inflammatory responses. In this process, IL-10 acts primarily as an inhibitor of the nuclear factor kappa B (NF-κB), and activator of transcription 1 (STAT1) signaling pathways (112, 113). Recent studies showed that once loss of IL-10, LPS could elicit higher tau hyperphosphorylation, neurotoxicity and IL-6 overexpression in mice, thereby leading to aggravation of insults to animals (114). Regarding up-regulation, compelling evidence reveals that Curcumin, an efficient pharmacological component of turmeric, has been proved to protect against neuronal loss by increasing the production of IL-10 in glial cells *via* the PI3K pathway (94) (**Figure 3**). Moreover, as a kind of anti-inflammatory leukocyte that secretes IL-10, the development of regulatory T cells (Tregs) may be aided by IL-10 (115). Therefore, IL-10 is of paramount importance in targeting therapy for neurodegenerative diseases.

IL-33, a branch of the IL-1 family and a kind of pro-inflammatory protein, is structural homology with IL-1 family cytokines. Similar to IL-1, IL-33 can be cleaved *in vitro* by caspase−1, generating an N−terminal fragment that is slightly shorter than the C−terminal fragment. IL-33 can be sequestered and blocked by soluble tumorigenicity 2 (sST2), an IL-33 decoy receptor, thereby curtailing pro-inflammatory response. However, numerous studies reveal a higher amount of serum sST2 in patients with mild cognitive impairment, suggesting that impaired IL-33/ST2 signaling may contribute to the pathogenesis of AD. Intriguingly, the exogenous administration of IL-33 has been shown to increase microglial recruitment and Aβ phagocytic activity *via* activating membrane-bound ST2 receptors and their downstream p38. This downstream cascade results in the suppression of pro-inflammatory genes and the polarization of microglia into anti-inflammatory phenotypes, which contributes to the secretion of the enzyme arginase 1 (ARG1) and the found of inflammatory zone 1 (FIZZ1) (116, 117). To sum up, anti-inflammatory cytokines are potential therapeutic agents for AD patients.

#### 4.2.3 Transforming Growth Factor β

The transforming growth factor-β (TGF-β) belongs to an evolutionarily conserved cytokine superfamily, and acts as cellular switches that regulate processes such as immune function, proliferation and epithelial−mesenchymal transition. Alternatively, it is closely pertinent to the cell differentiation and apoptosis (118). Notably, TGF-β deficiency in mice can lead to loss of microglia and the absence of a typical ramified morphology of microglia, indicative of a milieu molecule for TGF-β required for microglia function (119). In addition, TGF-β also regulates microglial homeostatic molecular and functional signature in the brain. It antagonizes specific cytokines such as IL-1 to inhibit both Th1 and Th2 reactions, preventing these cytokines from pro-inflammation in immune responses (120). Notably, TGF-β also functions as neurotrophic factor-promoting effects including differentiation, neuronal function maintenance, synapse plasticity, and memory formation (121). More importantly, TGF-β released primarily by microglia in response to CNS lesions protects neurons from toxins, ischemia, and Aβ aggregates, particularly *via* the phosphatidylinositol-3-kinase pathway in AD (122). Additionally, TGF-β helps to attenuate microglia clustering at neuritic plaques through SMAD2 phosphorylation and down-regulation of CCL5, ameliorating to the neuroinflammation caused by microglia in AD. As a result, the concomitant release and production of pro-inflammatory mediators and reactive oxygen species as critical contributing factors in AD pathology were further abrogated in the overactivated microglia (123). On the basis of an interesting study, 1,7-bis (4-hydroxy-3-methoxyphenyl) heptane-3,5-dione (tetrahydrocurcumin, THC) enhances the secretion of TGF-β in APP/ps1 mice. Subsequently, the study revealed that Aβ-induced reduced cell viability, cell cycle arrest and spatial memory impairment were rescued in mice by delivery of THC (124). Therefore, TGF-β may provide insights into microglial biology and the possibility of targeting microglia for the treatment of AD (**Figure 3**).

### 4.3 Microglial Phagocytosis, Autophagy and Aβ Degradation

As previously stated, AD is characterized as the deposition of Aβ and NFTs; thus, increasing microglial phagocytosis and autophagy to remove the overproduction of neurotoxic proteins is likely to be a promising treatment strategy.

#### 4.3.1 Scavenger Receptors Family

The SRs family was first described as a high-affinity, trypsin-sensitive, surface binding site of acetylated low-density lipoprotein (LDL) on macrophages of patients (125). Although the SRs family was initially considered to be closely related to lipid metabolism, its role in AD has been established, particularly for scavenger receptor class A (SR-A), CD36 and receptor for advanced end glycation products (RAGE) (30). Therefore, the critical molecules will be systematically reviewed as follows.

##### 4.3.1.1 Scavenger Receptor Class A

SR-A belongs to a large family of scavenger receptors consisting of at least 6 classes, all mediating the uptake of modified LDL. Until now, SR-A has been shown to be important in the inflammatory response in host defense, cellular activation, adhesion, and cell-cell interaction. An interesting recent study found that although SR-A and CD36 were the main receptors responsible for Aβ clearance, SR-A rather than CD36 mediated oligomeric Aβ internalization *via* adhesion to Aβ coated surfaces, and that the oligomeric Aβ was completely degraded within 30 minutes by cathepsin B, a cysteine protease in lysosomes (126). Therefore, the deficiency of SR-A causes the reduced uptake of Aβ. More than that, the decreased expression of SR-A is conducive to release of pro-inflammatory cytokines in the CNS, and reduces anti-inflammatory cytokines in plasma, which increases the inflammatory environment (127). In contrast, there is also evidence that SR-A plays a critical role in innate immune responses like inflammatory responses by combining with exotic pathogens or endogenous ligands, resulting in M1 polarization and secretion of ROS. As a result, the brain tissue may be damaged by pro-inflammatory cytokines due to overexuberant immune responses (128). Unfortunately, the drugs targeting SR-A of microglia was not hitherto discovered for AD treatment. Nevertheless, we are still optimistic about seeking an ideal target drug of molecular therapy for AD in the future.

##### 4.3.1.2 CD36

CD36, a membrane protein also known as SR-B2, is enriched in the monocyte-macrophage system and endothelial cells. As a member of SRs family, CD36 facilitates fatty acid uptake and intracellular trafficking of cholesterol for packaging into lipoproteins, which independently regulate the secretion of Aβ and tau *via* a druggable CYP46A1 (cholesterol 24-hydroxylase) -CE (cholesteryl esters) -tau axis (129, 130). Kunjathoor et al. demonstrated that Aβ promoted the accumulation of pro-inflammatory lipid peroxides by inhibiting CD36. This state will form vicious circle in AD, implying the aggravation of AD. In addition, CD36 is involved in mediating inflammatory processes by regulating macrophage migration, phagocytosis, and the formation of foam cells (131). Along with this, CD36 can also recognize Aβ and trigger Toll-like receptor 4 (TLR4)-TLR6 signaling, which shares the same route as activator oxidized low density lipoprotein (oxLDL). Strikingly, TLR4-TLR6 signaling could promote the mRNA encoding of pro-inflammatory cytokines like pro-IL-1β, thereby priming the activation of inflammasome and the secretion of nitric oxide and ROS. Accordingly, the disruption of CD36-TLR4-TLR6 signaling in microglia can effectively protect neurons against inflammatory toxicity resulting from Aβ (132). Nevertheless, another study found that CD36 can be up-regulated by a selective PPAR agonist, DSP-8658, then leading to accumulation of microglia in and around Aβ plaques and an increase in microglial Aβ phagocytosis. This event was verified by the rescue of spatial learning in APP/PS1 transgenic mice (133). On the other side, the upregulation of CD36 can alleviate microglia phagocytosis deficiency and spatial learning impairment of AD mice by intranasal administration of a kind of lentiviral vector encoding human nuclear factor erythroid 2-related factor 2 (LV-*NRF2*) which is a transcriptional activator to up-regulate CD36 expression (134). In general, AD therapeutic strategies targeting CD36 have two main focuses: CD36 blockade with neutralizing antibodies or other small molecules to suppress the inflammatory response of microglia, and upregulating the expression of CD33 to lower the Aβ burden, respectively. The exact mechanisms involved and specific equilibrium points between the two approaches have yet to be investigated.

##### 4.3.1.3 Receptor for Advanced End Glycation Products

RAGE is a member of the immunoglobulin superfamily of cell surface molecules. As a pattern-recognition receptor, it acts as a pro-inflammatory mediator by pairing with its ligands including advanced glycation end products (AGE), high mobility group box 1 (HMGB1) and Aβ oligomers. Critically, it also mediates plasma Aβ across blood brain barrier (BBB). Given that HMGB1-RAGE axis triggers pro-inflammatory microglia activation through RAGE-NF-κB signaling pathway, targeting the HMGB1/RAGE/NF-κB signaling pathway may be a potential strategy for the treatment of AD (135). Likewise, the activated microglia enhance the secretion of RAGE ligands, leading to neuronal cell oxidative stress and death in rats or mice and creating a vicious cycle as well (136). Soluble RAGE (sRAGE), a decoy receptor of AGE that lacks the transmembrane domain, may participate in conservation of CNS homeostasis by dint of blocking the combination of RAGE and AGE based on its well contributions to the removal/neutralization of circulating AGE ligands (137). Subsequently, evidence for an important role of RAGE from a study demonstrated that, a flavoring agent vanillic acid (VA), could antagonize RAGE-mediated c-Jun N-terminal kinase (JNK). It exhibits a number of attractively biological activities including anti-inflammatory, antioxidant and neuroprotection. Of importance, VA treatment attenuates synapse loss and memory deficits of AD markers like APP, BACE1 and Aβ induced by LPS (138). Notably, non-specific neuroinflammation and M1 polarization could be suppressed *via* inhibiting RAGE-NF-κB signaling pathway, implying VA potential as a therapeutic agent to target AD (139). Regarding involvement of RAGE in AD target therapy, there is now evidence that Aβ dependent impairments like dependent behavior and damaged dendritic spine morphology in the entorhinal cortex (EC) are rescued in transgenic mice with silenced RAGE of microglia *via* prohibiting kinases JNK and p38 mitogen-activated protein kinase (p38MAPK). Alternatively, the EC, a brain area crucially involved in learning and memory, is one of the most susceptible to neurodegenerative disorders such as AD due to the vulnerability of superficial neurons and its number reduces significantly in the early stage of AD. Therefore, inhibiting the RAGE of microglia in EC is likely to be another sensitive therapeutical target in AD (**Figure 3**) (140).

#### 4.3.2 Peroxisome Proliferator Activated Receptor

**Figure 4:**
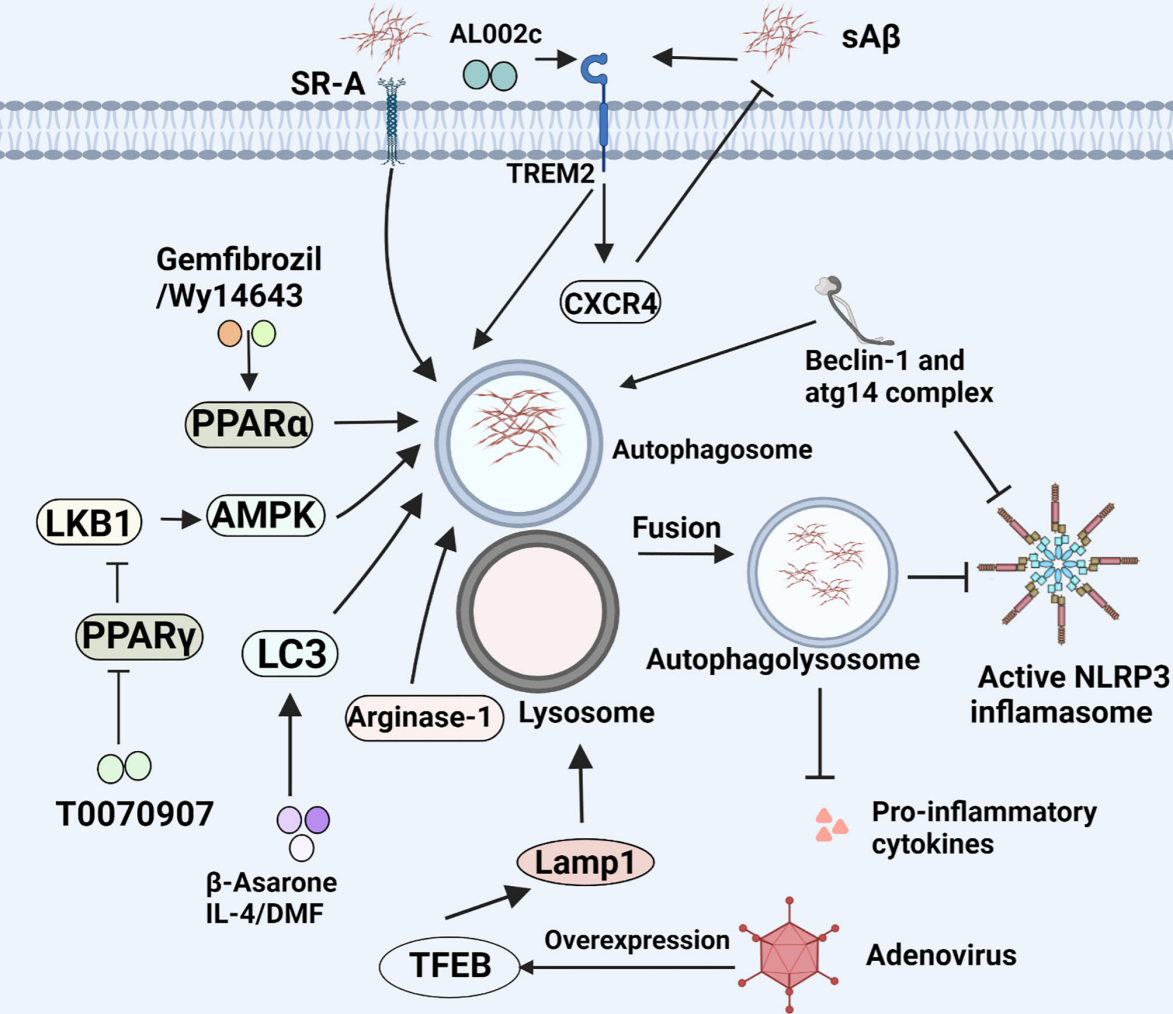
Microglial phagocytosis and autophagy in AD. Microglia eliminate Aβ load through phagocytosis and autophagy. This figure displays the regulatory mechanism of this process.

PPAR is a type of nuclear receptor that collaborates with PPAR response elements in the promoter region of genes to regulate glucose, lipid metabolism, as well as inflammatory processes. It has been proved that PPARα promotes the recruitment of microglia, and is closely associated with microglial Aβ phagocytosis. Nowadays, PPARα activation by its agonists Gemfibrozil and Wy14643, can improve a variety of pathological and behavioral phenotypes of AD. PPARα agonists showed an additive enhancement of the autophagy of microglia and structural neuroplasticity in APP-PSEN1ΔE9 mice (141). Therefore, PPARα activators that efficiently cross the blood–brain barrier may be considered as future therapeutics for AD. However, PPARγ appears an opposite effect. PPARγ antagonists can result in an overall reduction of Aβ levels and improved spatial memory performance. Evidence indicates that PPARγ antagonist T0070907 promotes microglia autophagy *via* Liver kinase B1 (LKB1)-adenosine 5’-monophosphate-activated protein kinase (AMPK) signaling pathway (**Figure 4**). Intriguingly, whether M1 to M2 polarization is involved in the process as well should be also concerned (142).

#### 4.3.3 Microtubule-Associated Protein Light Chain 3

LC3 is a component of the autophagosomal membrane, characterized as an autophagy marker, and primarily participates in the formation of autophagosomes. Autophagy-related gene 4 (ATG4) is essential for autophagy by affecting full length LC3 (pro-LC3) to the soluble form LC3-I. In general, LC3 is cleaved at its C-terminus by ATG4 to form LC3-I which is distributed in the cytosol. For autophagy, LC3-I covalently binds to phosphatidylethanolamine and is converted to LC3-II on autophagosome membranes. This process is mediated by the ATG12-ATG5-ATG16L complex, followed by the formation of autophagy precursors (143). The expression of LC3 proteins could be upregulated when nuclear factor erythroid 2-related factor 2 (NRF2), a factor is dissociated from Kelch-like ECH-associated protein 1 (KEAP1), and then translocates into the nucleus and binds to antioxidant responsive element (ARE) (144). There have been evidence that LC3-associated endocytosis (LANDO) not only reduces the deposition of Aβ and tau pathology, but also inhibits the secretion of inflammatory cytokines in activated microglia induced by Aβ. Concurrently, it also rescues memory and behavioral impairment of 5xFAD mice by maintaining neuronal homeostasis (145). In addition, the β-Asarone has been shown to promote autophagy by virtue of increasing the expression of LC3-I and LC3II (**Figure 4**) (146). Consistent with the finding, IL-4 is found to rapidly increase the expression of LC3-I and LC3II in BV2 microglia and increase the uptake and degradation of Aβ. But the inhibition of IL-4-pretreated microglia M1 phenotype switching induced by Aβ is independent on this autophagy (147). Along with the above-mentioned, use of some anti-inflammatory drugs like dimethyl fumarate (DMF), a potent Nrf2 activator may be considered as a potential therapeutic strategy by up-regulating LC3 to promote autophagy of microglia (148).

#### 4.3.4 Insulin Degrading Enzyme

Currently, pharmacological activation of the Aβ-degrading molecules represents a novel therapeutic strategy for the treatment of AD. Extracellular and intracellular degradation of Aβ depends mainly on two proteases, namely, neprilysin and insulin degrading enzyme (IDE) *in vitro* (149). IDE is a downstream region of insulin receptor signaling and mainly distributed in cytosol. The transport outside cells of IDE mainly relies on exosomes and extracellular vesicles. Compelling studies showed that decreased activity of any of these enzymes due to genetic mutation may increase the risk for AD. Farris et al. verified that mutations in *IDE* increased risk of developing AD in type 2 diabetes (T2D) (150). It is speculated that insulin resistance and overexpression compete with Aβ binding to IDE, resulting in IDE devitalization, which is responsible for impaired Aβ degradation and clearance in brain, and thereby promotes the pathogenesis of AD. Even more to the point, a slight overexpression of IDE remarkably alleviates Aβ deposition to a large extent in APP transgenic mice, implying that IDE may be an effective therapeutic target for AD (151). Statins including lovastatin and simvastatin which act as inhibitors of 3-hydroxy-3-methylglutaryl-coenzyme A (HMG-CoA) reductase (HMGR), play an important role in lowering serum cholesterol levels and are extensively used in the treatment of hypercholesterolemia (152). Surprisingly, Walter et al. found that lovastatin can stimulate the secretion of IDE in mouse microglia cell lines (BV-2 and N9 cells) *in vitro*, and the level of IDE in serum is significantly elevated following the delivery of lovastatin to mice as well (153). There is some evidence that somatostatin stimulates microglia to express and secrete IDE too (154). Apart from lovastatin, metformin, a derivative of biguanide, has been widely used for the treatment of type 2 diabetes (T2D) for almost all country. Intriguingly, an interesting study revealed that metformin, could also be a potential therapeutic agent for AD since it accelerates the expression of IDE by the activation of the AMPK signaling pathway (155). Consistently, administrating metformin orally also improves Aβ deposition, oxidative stress, and learning and memory functions in APP/PS1 mice (156). In short, it is possible to propose a new strategy for the targeted IDE-based therapeutical target for AD (**Figure 2**).

### 4.4 M1 to M2 Switch

As mentioned above, the M2 phenotype of microglia plays a relatively beneficial role in the maintenance of neurological homeostasis. Thus, increasing the M2 polarization of microglial cells may be a potential strategy for treatment of AD. It is reported that when TAK-242, a specific inhibitor of toll-like receptor 4 (TLR4), is administered, the levels of M1-markers (TNF and iNOS) are markedly reduced while M2-markers (TREM-2 and Arg-1) are increased conversely. In addition, TAK-242 potentiates microglial phagocytosis *via* the MYD88/NF-κB/NLRP3 signaling pathway, and it is capable of promoting Aβ clearance and inhibiting tau hyperphosphorylation, while it effectively ameliorates the learning and memory impairments (157). Besides, resveratrol is a natural polyphenolic phytochemical with a variety of bioactivities associated with health promotion. It effectively promotes microglia polarization into the M2 phenotype *via* proliferator-activated receptor coactivator-1 (PGC-1), while inhibiting NF-κB and activating STAT3 and STAT6 to increase M2 markers (158). Moreover, some non-drug treatments, for example, low-dose ionizing radiation (LDIR), may also affect the phenotype of microglia, increase TREM2 and CD206 expression in LPS-induced BV2 microglial cells, and attenuate Aβ deposition and cognitive decline ultimately (159). In addition, the LKB1–AMPK signaling pathway is not only involved in energy homeostasis, metabolic stress, but mediates M1 to M2 switch. Similar to drug, T0070907, an agonist of the peroxisome proliferator-activated receptor γ (PPARγ), also triggers M1-to-M2 polarization and abrogates the inhibition of LPS-induced autophagy by suppressing LKB1–AMPK signaling pathway (142). Other substances with similar effects on microglia polarization have been identified including L-cysteine-derived hydrogen sulfide (H2S) and CaMKK inhibitor, STO-609 (160, 161). Apart from that, several signaling pathways including mTOR, Rho/Rho-kinase, and the NOTCH signaling pathways, also involved in microglia polarization, implying a plethora of potential therapeutic targets in microglia polarization of AD (162–164). Numerous studies have shown that M2 polarization also helps to alleviate persistent neuropathic pain (NP) and AD-related behavior impairment in a doxycycline-induced mouse AD model (165). These evidences presented above demonstrate the significance of M2 polarization for the treatment of AD.

## 5 Conclusions and Future Expectations

In summary, microglia are crucial mediator and effector in the pathology of AD, but a slew of mysteries surrounding the interactions between microglia and AD remain unsolved. Neurodegeneration has been linked to microglia-associated inflammatory factors such as TNF-α, IFN-γ, and IL-1β. In this state, microglia fail to endocytose pathological Aβ and tau, and Aβ and tau deposition contributes to inflammatory activation, resulting in a vicious cycle in AD pathology. On the contrary, anti-inflammatory factors secreted by microglia such as IL-2, IL-4, IL-10 and TGF-β, and activation of certain receptors such as TREM2, aid in the restoration of learning and memory deficits in AD *via* various signaling pathways and mechanisms. Furthermore, phagocytosis and autophagy of microglia mediated by some critical receptors such as SR-A and CD36 are responsible for the degradation of deposited Aβ and tau in AD. The detailed potential interaction mechanism underlying a variety of molecular events orchestrated by microglia in AD is seen in figures mentioned above and **Table 1**. In the CNS, we believe that microglia, an obligatory member of the innate immune system, act as a protective factor against tumors, pathogens, and abnormally deposited proteins such as Aβ and tau. Notwithstanding, once Aβ and tau deposition exceeds that of clearance by microglia, the resulting excessive activation of microglia would release excessive pro-inflammatory factors to compromise neurons and their synapses associated with AD. In this review, we systemically summarized the microglia-associated factors, mechanisms of molecular activity, and relevant therapeutic strategies for AD that have been trialed in cell, animal models, or Alzheimer’s patients. More research has revealed that microglia play a critical role not just in AD, but also in PD, ischemia, demyelinating disease, and even psychiatric conditions such as mood disorders. Pro-inflammatory cytokines such like TNF-α and IL-6 have been shown to be increased in patients with depression, and human volunteers were indeed induced anxiety and depression by the treatment with pro-inflammatory cytokine IFN-α. Drug-induced anti-inflammatory microglial phenotypes have been shown to alleviate depressive-like behavior in mice (166). Taking into account all the great advantages of microglial double-edged sword defenses previously described, there is no doubt that targeting expansion of the microglial various beneficial bio-functions will hold the potential to delay disease onset and further possibly preserve cognitive function of patient with AD. Once we achieve great therapeutic outcomes, the therapeutic strategy targeting microglia would represent a perspective strategy for suppression of the early stage of neuropathological change in AD, and avoid the family tragedy of end-stage AD.

**Table 1:** Diverse drugs, their effects and associated signal pathways.

Drugs	Targets	Effects	Receptors	Effects	Pathways	Subjects
Galantamine	Cholinergic neuron	Activate	Cholinesterase	Inhibit	-	Cells/mice
OLT1177	NLRP3 Inflammasome	Inhibit	Caspase-1	Inhibit	-	Patients/mice
Stavudine	NLRP3 Inflammasome	Inhibit	-	-	AKT	Cells
JC-124	NLRP3 Inflammasome	Inhibit	Caspase-1	Inhibit	-	Mice
PF-04418948	Prostaglandin E2	Inhibit	EP-2	Inhibit	AKT	Cells
Indomethacin	Aβ-42	Reduce	γ-secretase	Switch	-	Cells
SEO	IL-1β	Inhibit	P38	Inhibit	NF-κB	Mice
Glycine	IFN-γ	Inhibit	-	-	-	Cells
RhinacanthinC	IFN-γ	Inhibit	-	-	NF-κB	Cells
APN	TNF-α	Inhibit	AdipoR1	Activate	AMPK-NF-κB	Cells
AL002c	TREM2	Activate	anti-hTREM2 agonistic	Activate	ITAM	Patients/mice
curcumin	IL-10	Activate	-	-	PI3K	Rats
THC	TGF-β	Activate	-	-	Ras/ERK	Cells
DSP-8658	CD36	Activate	PPARγ	Activate	-	Mice
V.A	RAGE	Inhibit	-	-	JNK	Mice
Gemfibrozil/Wy14643	PPARα	Activate	-	-	-	Mice
Metformin	IDE	Activate	-	-	AMPK	Mice
TAK-242	M1	M1 to M2 switch	TLR4	Inhibit	MyD88/NF-kappaB/NLRP3	Mice
Resveratrol	M1	M1 to M2 switch	PGC-1α	Inhibit	NF-κB/STAT	Cells

## Author Contributions

YC and JL wrote the manuscript with support from BW, MS, and HY. All authors contributed to the article and approved the submitted version.

## Funding

This work was supported by grants from the National Key R&D Program of China (2019YFA0802600), the National Natural Science Foundation of China (81974244, 82071551, and 81570960), and the Postgraduate Research & Practice Innovation Program of Jiangsu Province (5832013521).

## Conflict of Interest

The authors declare that the research was conducted in the absence of any commercial or financial relationships that could be construed as a potential conflict of interest.

## Publisher’s Note

All claims expressed in this article are solely those of the authors and do not necessarily represent those of their affiliated organizations, or those of the publisher, the editors and the reviewers. Any product that may be evaluated in this article, or claim that may be made by its manufacturer, is not guaranteed or endorsed by the publisher.
